# Safeguarding human–wildlife cooperation

**DOI:** 10.1111/conl.12886

**Published:** 2022-06-09

**Authors:** Jessica E. M. van der Wal, Claire N. Spottiswoode, Natalie T. Uomini, Mauricio Cantor, Fábio G. Daura‐Jorge, Anap I. Afan, Mairenn C. Attwood, Jenny Amphaeris, Fatima Balasani, Colleen M. Begg, Cameron J. Blair, Judith L. Bronstein, Iahaia O. Buanachique, Rion R. T. Cuthill, Jewel Das, Apurba Deb, Tanmay Dixit, Gcina S. Dlamini, Edmond Dounias, Isa I. Gedi, Martin Gruber, Lilian S. Hoffmann, Tobias Holzlehner, Hussein A. Isack, Eliupendo A. Laltaika, David J. Lloyd‐Jones, Jess Lund, Alexandre M. S. Machado, L. Mahadevan, Ignacio B. Moreno, Chima J. Nwaogu, Valdomiro L. Pereira, Raymond Pierotti, Seliano A. Rucunua, Wilson F. dos Santos, Nathalia Serpa, Brian D. Smith, Irina Tolkova, Tint Tun, João V. S. Valle‐Pereira, Brian M. Wood, Richard W. Wrangham, Dominic L. Cram

**Affiliations:** ^1^ FitzPatrick Institute of African Ornithology, Department of Science and Innovation‐National Research Foundation Centre of Excellence University of Cape Town Cape Town South Africa; ^2^ Department of Zoology University of Cambridge Cambridge UK; ^3^ Max Planck Institute for Evolutionary Anthropology Leipzig Germany; ^4^ Department of Ecology and Zoology Universidade Federal de Santa Catarina Florianopolis Brazil; ^5^ Department of Fisheries, Wildlife and Conservation Sciences, Marine Mammal Institute Oregon State University Corvallis Oregon USA; ^6^ Department for the Ecology of Animal Societies Max Planck Institute of Animal Behavior Radolfzell Germany; ^7^ Centre of Marine Studies Universidade Federal do Paraná Curitiba Brazil; ^8^ A.P. Leventis Ornithological Research Institute University of Jos Jos Nigeria; ^9^ School of Arts, Culture and Language Bangor University Bangor UK; ^10^ Mbamba Village Niassa Special Reserve Mbamba Mozambique; ^11^ Niassa Carnivore Project TRT Conservation Foundation Cape Town South Africa; ^12^ Department of Ecology & Evolutionary Biology University of Arizona Tucson Arizona USA; ^13^ Institute of Marine Sciences University of Chittagong Chittagong Bangladesh; ^14^ Department of Conservation and Climate Government of Manitoba Winnipeg Manitoba Canada; ^15^ Mlindazwe Lavumisa Shiselweni Kingdom of Eswatini; ^16^ CEFE Univ Montpellier, CNRS, EPHE, IRD Montpellier France; ^17^ Northern Rangeland Trust Isiolo Kenya; ^18^ Department of Anthropology and Cultural Research University of Bremen Bremen Germany; ^19^ Cytogenetics and Evolution Lab, Instituto de Biociências Universidade Federal do Rio Grande do Sul Porto Alegre Brazil; ^20^ Seminar für Ethnologie Martin‐Luther‐University Halle‐Wittenberg Halle Germany; ^21^ Kivulini Trust Kitengela Kenya; ^22^ Ngorongoro Conservation Area Authority Ngorongoro Tanzania; ^23^ Department of Physics Harvard University Boston Massachusetts USA; ^24^ School of Engineering and Applied Sciences Harvard University Cambridge Massachusetts USA; ^25^ Department of Organismic and Evolutionary Biology Harvard University Cambridge Massachusetts USA; ^26^ Centro de Estudos Costeiros, Limnológicos e Marinhos Universidade Federal do Rio Grande do Sul Imbé Brazil; ^27^ Programa de Pós‐Graduação em Biologia Animal Universidade Federal do Rio Grande do Sul Porto Alegre Brazil; ^28^ Tramandaí Inlet, Tramandaí, Rio Grande do Sul, southern Brazil Brazil; ^29^ Department of Ecology & Evolutionary Biology University of Kansas Lawrence Kansas USA; ^30^ Praia da Tesoura Laguna Brazil; ^31^ Wildlife Conservation Society Bronx New York USA; ^32^ Sanchaung Yangon Myanmar; ^33^ Department of Anthropology University of California, Los Angeles Los Angeles California USA; ^34^ Department of Human Behavior, Ecology, and Culture Max Planck Institute for Evolutionary Anthropology Leipzig Germany; ^35^ Department of Human Evolutionary Biology Harvard University Cambridge Massachusetts USA

**Keywords:** animal culture, biocultural conservation, biodiversity conservation, dolphins, honeyguides, human–wildlife interactions, interspecies cooperation, mutualism, orcas, wolves

## Abstract

Human–wildlife cooperation occurs when humans and free‐living wild animals actively coordinate their behavior to achieve a mutually beneficial outcome. These interactions provide important benefits to both the human and wildlife communities involved, have wider impacts on the local ecosystem, and represent a unique intersection of human and animal cultures. The remaining active forms are human–honeyguide and human–dolphin cooperation, but these are at risk of joining several inactive forms (including human–wolf and human–orca cooperation). Human–wildlife cooperation faces a unique set of conservation challenges, as it requires multiple components—a motivated human and wildlife partner, a suitable environment, and compatible interspecies knowledge—which face threats from ecological and cultural changes. To safeguard human–wildlife cooperation, we recommend: (i) establishing ethically sound conservation strategies together with the participating human communities; (ii) conserving opportunities for human and wildlife participation; (iii) protecting suitable environments; (iv) facilitating cultural transmission of traditional knowledge; (v) accessibly archiving Indigenous and scientific knowledge; and (vi) conducting long‐term empirical studies to better understand these interactions and identify threats. Tailored safeguarding plans are therefore necessary to protect these diverse and irreplaceable interactions. Broadly, our review highlights that efforts to conserve biological and cultural diversity should carefully consider interactions between human and animal cultures.

Please see AfricanHoneyguides.com/abstract‐translations for Kiswahili and Portuguese translations of the abstract.

## 1 INTRODUCTION

Conservation decision makers routinely address conflict between human interests and the protection of wildlife and ecosystems (Dickman, 2010), yet challenges remain even when the interests of humans and wildlife are aligned. Among human–wildlife mutualisms—reciprocally beneficial interactions between humans and free‐living, wild, nonhuman animals (Dounias, 2018)—we find few remaining forms of “human–wildlife cooperation,” in which individuals of both species actively cooperate with each other in a coordinated manner to achieve a mutually beneficial outcome (Box 1). This definition specifies cooperative behavior with a wild animal, and we therefore exclude other human–wildlife mutualisms in which benefits are received indirectly through a regulating ecosystem service (e.g., scavenging of carrion and waste, controlling pests), and where the animal is coerced or from a domesticated lineage (see Dounias, 2018). All examples currently known to science involve cooperative foraging (Box 1), but as‐yet undescribed forms could confer different types of benefits (Cram et al., in press).

**BOX 1: The diversity of human–wildlife cooperation**

Full list of citations in Table S1.

**Human–honeyguide cooperation**

In parts of Africa, humans cooperate with greater honeyguides (*Indicator indicator*) to gain access to the content of African honeybee (*Apis mellifera scutellata* and *A. m. capensis*) nests (Isack & Reyer, 1989) and sometimes meliponine stingless bee species (Spottiswoode et al., 2016). Human “honey‐hunters” attract greater honeyguides with loud sounds that can include calling, shouting, whistling, and/or banging tools against trees (Isack & Reyer, 1989; Laltaika, 2021; Spottiswoode et al., 2016; van der Wal et al., 2022). The honeyguide approaches and responds with a chattering call, then flies in the direction of a bees’ nests (Isack & Reyer, 1989). After the honey‐hunter subdues the bees and harvests the nest with fire and tools, the honeyguide feeds on beeswax left behind, supplementing its otherwise insectivorous diet. Of the 17 honeyguide species in Africa, only the greater honeyguide is confirmed to cooperate with humans (but similar cooperation has been suggested in other honeyguides species; see Table S2). There is no conclusive evidence that honeyguides cooperate with other species that forage on honey, such as honey badgers (*Mellivora capensis*) (Dean et al., 1990).

**FIGURE 1:**
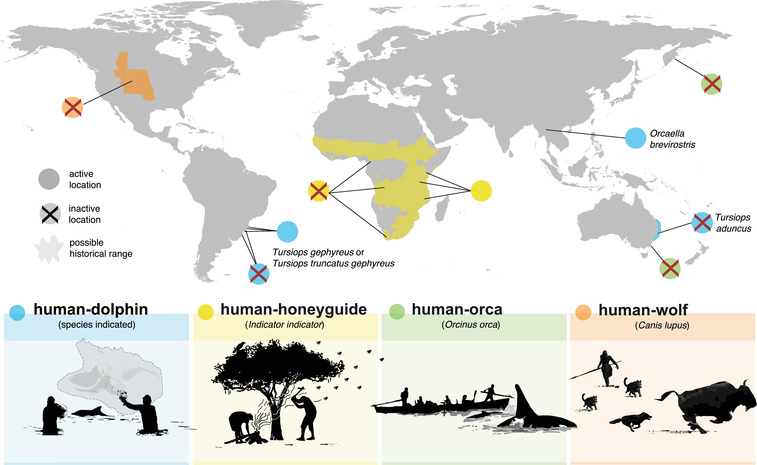
Active and inactive forms of human–wildlife cooperation known to the scientific community or reported in detail by Indigenous peoples, and their locations (see Table S1 for references and Table S2 for additional potential cases). Solid dots indicate active locations, crossed dots indicate inactive locations (i.e., where cooperation is currently absent, but there is strong evidence for its presence in the past), and shaded areas indicate possible historical ranges. Human–dolphin illustration: original art by M.C.; human–honeyguide illustration: inspired by J. Solomon; human–orca illustration: inspired by C.E. Wellings; human–wolf illustration: inspired by D. Eskridge

Human–honeyguide cooperation may have existed for hundreds of thousands of years: both honeybees and the greater honeyguide lineage existed in Africa when hominids gained control of fire approximately 1.5 m.y.a. (Gowlett, 2016; Wrangham, 2012). While cooperation was presumably once common throughout much of sub‐Saharan Africa, it has disappeared from many places in recent generations (Figure 1; Table S1). Among the few remaining areas where people still heavily rely on wild honey and on greater honeyguides to find bees’ nests, human cultures vary in traits relevant to the honeyguides.

Honey‐hunters mostly learn to honey‐hunt from their fathers (Laltaika, 2021; Spottiswoode et al., 2016; van der Wal et al., 2022; Wood et al., 2014), but horizontal transmission also operates. In honeyguides, guiding behavior is likely partly innate, but further reinforced and refined through learning in environments in which humans cooperate. Honeyguides learn to respond appropriatelyto local cultural variation in human signaling (C.N.S. and B.M.W., unpublished data), which is remarkable given that honeyguides are brood parasites (raised by other bird species) and therefore cannot rely on social information from their parents. Ongoing research is focusing on whether honeyguides learn socially (horizontally), or whether guiding behavior is only individually learnt.

**Human–dolphin cooperation**

Cooperation between fishers and dolphins exists or has recently existed in seven populations: at five sites in southern Brazil (three active, two inactive) with Lahille's bottlenose dolphins (*Tursiops truncatus gephyreus* or *Tursiops gephyreus*, ongoing taxonomic debate), one active site in the Ayeyarwady river in Myanmar with Irrawaddy dolphins (*Orcaella brevirostris*), and one inactive site on the eastern Australian coast with Indo‐Pacific bottlenose dolphins (*Tursiops aduncus*) (Figure 1; Table S1; even though orcas are taxonomically Delphinids, we discuss them separately in the next section, due to significant differences in the nature of their interactions). In all cases, human–dolphin cooperation involves interspecies coordination to access a variety of fish species, which in most cases are migratory mullet (Mugilidae). There are several other locations where apparently positive, likely mutualistic human–dolphin interactions take or have taken place (Table S2), but lack sufficient evidence for cooperation, either because the benefit for the dolphin is yet to be quantified or because the interaction does not involve active, simultaneous human–dolphin coordination. Although it is challenging to obtain precise behavioral observations, the main role played by dolphins seems to be detecting fish and aggregating their schools toward shallow waters where the fishers deploy fishing gear to disrupt fish schools (standing on the shore or in boats; Simões‐Lopes et al., 1998; Smith et al., 2009; Tun, 2014). Fishers await specific behaviors by the dolphins, which they perceive as signals, before deploying their nets (Fairholme, 1856; Serpa, 2019; Simões‐Lopes et al., 1998; Smith et al., 2009). In some of the interactions, fishers use acoustic signals such as tapping on the side of their vessel with a conical wooden pin, or slapping the water surface with the flat end of their paddle (Busnel, 1973; Smith et al., 2009; Tun, 2004).

Dolphins socially learn how to cooperate with fishers, both vertically (from their mothers; Simões‐Lopes et al., 1998) and horizontally (from their peers; Daura‐Jorge et al., 2012; Simões‐Lopes et al., 2016). Dolphins that cooperate with humans prefer to associate socially with other cooperative dolphins (Machado, Cantor, et al., 2019), likely allowing them to learn socially and hone cooperative foraging skills. This is reflected in long‐term monitoring studies showing that juvenile dolphins can quickly adopt the skills needed to cooperate with fishers (M.C. & F.G.D.J., unpublished data). Some young fishers learn the core cooperative fishing skills from their close relatives, but others learn these core and additional skills through interactions with nonrelated fishers within the same or neighboring communities (da Rosa et al., 2020; Peterson et al., 2008; Silva et al., 2021). Fishers who learn the tradition through vertical transmission are better at recognizing individual dolphins and their stereotyped behaviors, and may be less prone to innovations or mistakes that could affect dolphins’ behavior (da Rosa et al., 2020).

**Human–orca cooperation**

Cooperation between humans and orcas (*Orcinus orca*; the world's largest Delphinids species) is known to have occurred in at least two locations in Russia and in Australia (Figure 1; Table S1). Orcas herded whales and other marine mammals to the surface or shoreline, where humans would kill the prey, and share it with the orcas (Clode, 2002). Cooperation between orcas and people in Australia is thought to have lasted nearly 100 years until it ceased when European settlers forced Indigenous people to move out of the area (Neil, 2002). There are three other locations where human–orca interactions were potentially cooperative, but detailed evidence is lacking (Table S2).

**Human–wolf cooperation**

Close interactions between Indigenous peoples and wolves (*Canis lupus)* were once common across North America (Barsh & Marlor, 2003; Pierotti & Fogg, 2017). Numerous Indigenous reports suggest that people and wolves cooperated to hunt bison (genus *Bison*) and other large prey. Scientific hypotheses propose that wolves were more efficient at tracking and chasing prey, while humans could kill the exhausted animal moreefficiently with spears (e.g., Shipman, 2015). The hunters involved left a share of the meat for the wolves (Pierotti & Fogg, 2017). However, detailed first‐hand accounts of human–wolf cooperation in the scientific literature are lacking, likely because of the reservations of some groups of Indigenous peoples about disclosing culturally sensitive information to outsiders, and because the interaction is extinct. However, remnants of positive interactions may persist in isolated locations (Nelson, 1983; Haber & Holleman, 2013; Pierotti & Fogg, 2017), and aspects of them may be evident in humans’ interactions with domesticated dogs. Most evidence consistent with human–wolf cooperation involves North America, but there are indications that cooperative human–wolf interactions potentially also happened in Europe and Asia (Table S2).

**Candidate forms of human–wildlife cooperation**

Corvids

There are numerous reports of crows and ravens being indicators of resource availability, but it is currently unclear whether these are cooperative interactions. For example, common ravens (*Corvus corax*) indicate the locations of ungulates or scavenging opportunities for hunters in North America and Europe (e.g., Heinrich, 1999). However, whether common ravens actively seek to attract humans, and whether they actively coordinate their behavior to achieve a mutually beneficial outcome, remains to be investigated. Another candidate form of human–wildlife cooperation requiring further investigation involves Kanak people and New Caledonian crows (*Corvus moneduloides*) in New Caledonia. People follow crows to candlenut trees (*Aleurites moluccana*) that harbor the highly nutritious larvae of a longhorn beetle (*Agrianome fairmairei*), that they use as fishing bait. After the tree is broken open by the human(s), crows have increased access to the larvae (N.T.U. personal observation). To determine whether this is an instance of human–wildlife cooperation, current research seeks to establish whether both parties actively attract each other's attention (N.T.U., unpublished data).

Other honeyguide species

In east‐central and central African rainforest where the greater honeyguide is apparentlyabsent, there are several reports that hunter‐gatherers cooperate with other honeyguide species (Indicatoridae) (Dounias, 2018), including dwarf honeyguides (*I. pumilio*) to access the content of stingless bees’ nests (Kajobe & Roubik, 2007). There are also anecdotal reports of scaly‐throated honeyguides (*I. variegatus*) guiding in other parts of Africa (Table S2), where greater honeyguides do occur. Key details of the putative cooperation, including the birds’ behavioral contribution to the interaction, remain to be investigated.

The active forms of human–wildlife cooperation currently known to the scientific community are human–honeyguide cooperation, in which honey‐hunters cooperate with birds called greater honeyguides (*Indicator indicator*, hereafter “honeyguides”) to access the content of bees’ nests (mostly African honeybees, primarily *Apis mellifera scutellata*), and human–dolphin cooperation, in which artisanal fishers cooperate with several delphinid species (hereafter “dolphins”) to catch fish (Box 1; Figure 1). They vary geographically: a mosaic of variably active human–honeyguide cooperation exists across sub‐Saharan Africa, while active human–dolphin cooperation occurs in isolated locations across southern Brazil and in Myanmar, and involves a small subset of dolphin populations of two species (Figure 1; Table S1). Several other cases of human–wildlife cooperation are inactive, including human–dolphin cooperation in Australia and at two sites in Brazil (Table S1), as well as human–orca cooperation in Australia and Russia (Neil, 2002), which involved cooperation between human whale‐hunters and orcas (*Orcinus orca*) to hunt marine mammals (Box 1; Figure 1). Human–wolf cooperation, in which Indigenous peoples and wolves (*Canis lupus*) coordinated while hunting large ungulates, is thought to have occurred in North America (Fogg et al., 2015; Pierotti & Fogg, 2017) (Box 1; Figure 1). Candidate forms of human–wildlife cooperation are outlined in Box 1.

Human–wildlife cooperation faces several current and anticipated threats, resulting in one or both partners losing the benefit of the interaction. They encounter unique conservation challenges, as the needs of multiple species must be considered, as well as the environment in which they function, and the knowledge of how to interact. In this review, we (i) outline the value of these rare, active cases of human–wildlife cooperation; (ii) describe the causes of their decline and loss; and (iii) provide priorities and recommendations for sustainable and tailored safeguarding.

## 2 SIGNIFICANCE OF HUMAN–WILDLIFE COOPERATION

### 2.1 Significance to humans

Human–wildlife cooperation provides both material and nonmaterial benefits to human partners and their communities. It provides increased access to important resources, such as nutritionally rich honey in human–honeyguide cooperation (Marlowe et al., 2014; Spottiswoode et al., 2016; Wood et al., 2014) and a variety of fish species (often mullets; Mugilidae) in human–dolphin cooperation (e.g., Simões‐Lopes et al., 1998; Smith et al., 2009; Tun, 2004). Cooperation with the wildlife partner significantly increases the quantity and quality of the resource harvested by the human, compared to similar small‐scale harvesting without help of the wildlife partner (Isack & Reyer, 1989; Santos et al., 2018; Simões‐Lopes et al., 1998; Smith et al., 2009; Tun, 2005; Wood et al., 2014). These resources contribute to subsistence, increase food security, and enable income or trade (Peterson et al., 2008; Smith et al., 2009; Spottiswoode et al., 2016; Tun, 2014; Wood et al., 2014). Honey from human–honeyguide cooperation is also used as medicine, in ceremonies, and to brew alcohol (Isack, 1987, 1999; Laltaika, 2021).

In many human communities that engage in human–wildlife cooperation, the interaction itself is also of cultural value (Clode, 2002; Fogg et al., 2015; Gruber & Sanda, 2019; Isack, 1987; Laltaika, 2021; Neil, 2002). Engaging in human–dolphin cooperation in Brazil, for example, provides nonmaterial benefits to fishers that include cultural belonging, a sense of place, leisure, and recreation (Machado, Daura‐Jorge, et al., 2019), and strengthening of affiliative and cooperative relationships among fishers (Santos‐Silva et al., 2022). In Cameroon, stories of human–honeyguide mutualism are an important aspect of rituals and oral history (Gruber & Sanda, 2019). Components of human–wildlife cooperation often form a “cultural complex” within a society (Sapir, 1916), meaning that an integrated set of practices and beliefs is structured around it, including ecological and cultural knowledge, folklore, ritualized attributes, and symbolic value. The wildlife partner can be a “cultural keystone species” in the human communities involved, contributing substantially to the local cultural identity (human–dolphin cooperation [Catão & Barbosa, 2018; Silva et al., 2021; Smith et al., 2009] and human–wolf cooperation [Dounias, 2018; Pierotti & Fogg, 2017]).

Additionally, human–wildlife cooperation has scientific value. Ancient forms of human–wildlife cooperation provide insights into the diverse ways our ancestors interacted with the natural world. Our own species comprises half of the partnership, providing an unusual opportunity to experimentally study cooperation since humans’ contribution can be manipulated in sensitively designed experiments (e.g., Spottiswoode et al., 2016).

### 2.2 Significance to biodiversity

Cooperation with humans appears to give wildlife increased access to important resources, such as bees’ wax and larvae for honeyguides (Isack & Reyer, 1989), and fish for dolphins (Simões‐Lopes et al., 1998; Smith et al., 2009). While precise benefits are yet to be quantified, honeyguides rarely have access to beeswax without human intervention (Isack & Reyer, 1989), and it is hypothesized that dolphins are more easily able to access fish schools disrupted by barriers or fishing gear (Simões‐Lopes et al., 1998; M.C. & F.G.D.‐J., unpublished data). Although the benefits to dolphins remain inconclusive due to the logistical difficulties of following them in turbid water, the fact that the dolphins often actively initiate the interaction suggests that doing so is beneficial (Neil, 2002; Simões‐Lopes et al., 1998; Smith et al., 2009; Tun, 2004). In one population of Lahille's bottlenose dolphins, cooperating with fishers is associated with increased survival, arising from reduced risk of bycatch for those that cooperate (Bezamat et al., 2021, 2018). Cooperation is also associated with stronger social relationships (Machado, Cantor, et al., 2019), and could broadly confer similar benefits to “play” behavior seen in many dolphin populations, by contributing to the dolphins’ physical, social, and emotional development and well‐being (Hill et al., 2017).

Local behavioral adaptations to human cooperation mean that wildlife partners may represent demographically or culturally distinct populations, thereby increasing biodiversity (Brakes et al., 2021). Animal cultures are broadly defined as behavioral repertoires that are socially learnt, shared within subsets of a population, and inherited nongenetically by successive generations (e.g., Laland & Hoppitt, 2003). Cooperative foraging behavior is most likely culturally transmitted in dolphins (Simões‐Lopes et al., 2016). In Laguna, Brazil, the social network of bottlenose dolphins is structured by their cooperation with artisanal fishers: there are different social communities of cooperative and noncooperative dolphins within the same population (Daura‐Jorge et al., 2012), which have distinct vocal communication (Romeu et al., 2017) and home ranges (Cantor et al., 2018). Honeyguides adapt at least behaviorally to variation in human traits (C.N.S. & B.M.W., unpublished data), perhaps facilitated by social learning (Spottiswoode et al., 2016). Social transmission of behaviours associated with human–wildlife cooperation in both systems generates the potential for cultural coevolution (Marzluff & Angell, 2005).

Human–wildlife cooperation also has wider ecological impacts. Other vertebrate species feed on beeswax made available by human–honeyguide cooperation (D.J.L.‐J. & C.N.S., unpublished data). Current research in the Niassa Special Reserve in northern Mozambique is investigating whether and how honey‐hunting may influence the wider ecosystem through its effects on bees, trees, and fire. Fishing with dolphins is a specialized small‐scale fishery, which has lower impacts on fish populations than other techniques, and results in very little or no bycatch (Bezamat et al., 2021; Zappes et al., 2011), promoting sustainability of fisheries. Ongoing research is investigating how increased rate of prey capture by both partners may influence population dynamics and movements of the fish species, directly and indirectly affecting community ecology.

## 3 WHAT THREATS IS HUMAN–WILDLIFE COOPERATION FACING?

**FIGURE 2:**
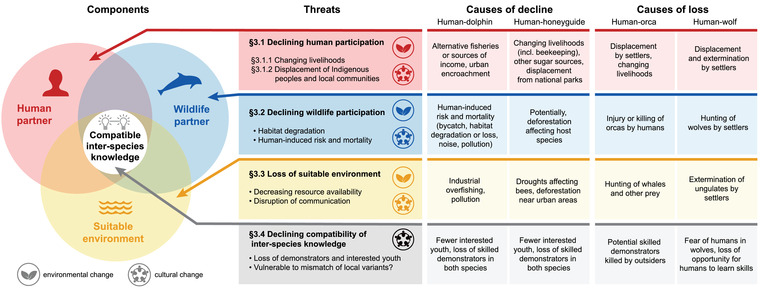
Human–wildlife cooperation faces particular conservation challenges, as it requires four components: (i) a motivated human partner, (ii) a motivated wildlife partner, (iii) a suitable environment, and (iv) compatible interspecies knowledge. We summarize the threats faced by each of these components (numbered according to sections in main text), whether these are driven by environmental and/or cultural change, and the causes of decline and loss for active and inactive forms of human–wildlife cooperation, respectively. See Table S3 for references

Four components are needed to maintain the active cases of human–wildlife cooperation: (i) a motivated human partner; (ii) a motivated wildlife partner; (iii) a suitable environment; and (iv) compatible interspecies knowledge (i.e., learnt information pertaining to species interaction) (Figure 2). Next, we review the environmental and cultural changes, and their interplay, known to pose threats to these components.

### 3.1 Declining human participation

#### 3.1.1 Changing livelihoods

Human engagement in human–wildlife cooperation may decline for environmental and/or cultural reasons. First, economic and social developments may provide new ways to obtain the resource, replacing cooperation. For example, apiculture or other sugar sources may become preferred over cooperating with honeyguides (Gruber, 2018; Gruber & Sanda, 2019; Isack, 1987, 1999), and alternative fishing techniques may become more efficient than cooperating with dolphins (Campredon & Cuq, 2001; Smith et al., 2009; Tun, 2004), although this remains to be quantified. Second, even where people remain committed to the cooperative partnership, wider economic changes can lead to reduced demand or availability of the resource, or to the displacement of motivated wildlife and human partners. For example, in Brazil, the sale price of mullet for artisanal fishers can be reduced if the commercial fishing industry oversupplies the local fishing market (Souza et al., 2017). In Myanmar, fishers using illegal electric fishing methods appear to have depleted local fish stocks and contributed to declines in local dolphin populations, and their aggressive behavior has discouraged local fishers from engaging in cooperation with dolphins (Smith et al., 2009; Thomas et al., 2019; Tun, 2014).

Human communities differ in their tendency to pursue alternative livelihoods, because these may be incompatible with their lifestyle or cultural values. For example, some honey‐hunting cultures appear to be more likely to shift toward apiculture than others. Likely social and cultural drivers of such shifts include the market demand for honey (Gruber & Sanda, 2019; Laltaika, 2021) and whether wild honey is more valued than honey from beehives, because of its medicinal or ceremonial value (Laltaika, 2021). Similarly, human cultures involved in human–dolphin cooperation vary in economic and cultural reliance on dolphins: professional fishers may be more likely to switch to more efficient fishing methods, compared to opportunistic or amateur fishers who seek cultural or social rather than livelihood benefits (Machado, Daura‐Jorge, et al., 2019).

#### 3.1.2 Displacement of Indigenous peoples and local communities

Forced removal of people from their lands—whether by agricultural settlers, land grabbers/speculators, urban developers, or conservationists—can threaten human engagement in human–wildlife cooperation. In Tramandaí, southern Brazil, urban development displaced fishing community neighborhoods to the city's periphery, far away from human–dolphin cooperative sites (Ilha et al., 2020). The view that “wilderness” is best conserved as a landscape without humans (“fortress conservation”) has in some places led to the extirpation of Indigenous connections with the ecosystem (Pierotti, 2011). Where fortress conservation prevents residents from living and foraging in national parks, benefits of human–wildlife cooperation to the wildlife partner, and the wider ecosystem, are lost. Fortress conservation has led to the local disappearance of human–honeyguide cooperation in parts of Africa (Dean et al., 1990; Isack & Reyer, 1989; Laltaika, 2021). Even where Indigenous and local communities have been granted access to protected areas, their role in ecosystem functioning is sometimes ignored, particularly when it does not align with a desire for “undisturbed” habitats (Borgerhoff Mulder & Coppolillo, 2005).

### 3.2 Declining wildlife populations and participation

Declining motivation by the wildlife partner to engage in human–wildlife cooperation is typically attributable to change in the human partner (§3.1) or the environment (§3.3). There are numerous anecdotal reports of honeyguide populations that no longer regularly guide humans in locations where humans ceased to respond to them multiple human generations ago (Dean et al., 1990; Friedmann, 1955; Isack, 1999). Increased commercial and illegal fishing activity (e.g., electric fishing and gillnets in Myanmar [Smith et al., 2009; Tun, 2014] and gill and trammel nets in Brazil [Peterson et al., 2008]) leads to more dolphin bycatch (Bezamat et al., 2021; Tun, 2014), and boat engine noise and chemical pollution can alter dolphins’ behavior (Pellegrini et al., 2021; Tun, 2014), health (Righetti et al., 2019), and survival (Bezamat et al., 2020). This not only decreases the subpopulation of cooperating dolphins, but often endangers the species (e.g., Bezamat et al., 2021; Tun, 2014). Both dolphin species involved in human–dolphin cooperation are threatened, with the Lahille's bottlenose dolphin listed as Vulnerable, and the Ayeyarwady River subpopulation of Irrawaddy dolphin in Myanmar considered Critically Endangered (IUCN, 2021). Laws against fishing practices that can kill dolphins are rarely enforced (Bezamat et al., 2021; Smith et al., 2009). Habitat disturbance (such as dredging and construction for coastal and riverine development) can change dolphins’ habitat use, leading to temporary or permanent abandonment of important areas, including where dolphins and fishers cooperate (Agrelo et al., 2019). While honeyguides are widespread across sub‐Saharan Africa and their conservation status is Least Concern (IUCN, 2021), they are brood parasites and so rely on other species to raise their young, making them vulnerable to any threats affecting their host species.

### 3.3 Loss of suitable environment

Environmental change (e.g., habitat degradation or human encroachment), often preceded by human cultural change (e.g., maritime or urban development), can threaten human–wildlife cooperation by decreasing resource availability. Poor governance, overfishing by larger fishing industries (Sant'Ana et al., 2017), and changes in mullet nursery grounds (de Abreu‐Mota et al., 2018) can threaten mullet stocks and availability at human–dolphin cooperative sites (e.g., Santos et al., 2018; Simões‐Lopes et al., 1998). Thriving bee populations are crucial for human–honeyguide cooperation, and although honeybee colonies are generally abundant in Africa (Pirk et al., 2016), some honey‐hunting communities report that bee populations have locally declined, likely due to increasingly frequent droughts (Laltaika, 2021; van der Wal et al., 2022), deforestation (Gruber & Sanda, 2019), and overharvesting owing to increased population pressure. Presence of people not engaged in human–dolphin cooperation can also threaten the interaction. Increased competition with unlicensed fishers and tourists who want to experience interaction with dolphins for fun can overcrowd cooperative sites in Brazil, reducing opportunities for safe interaction with dolphins (Silva et al., 2021). Boat noise can also interfere with dolphins’ acoustic communication and echolocation while cooperating with humans (Pellegrini et al., 2021). Similarly, the presence of other people (e.g., poachers, insurgents, refugees, or antipoaching scouts who misidentify honey‐hunters for poachers) in honey‐hunting habitats can pose a security threat to honey‐hunters, discouraging collection of wild honey (Gruber & Sanda, 2019; van der Wal et al., 2022).

### 3.4 Declining compatibility of inter‐species knowledge

Human–wildlife cooperation involves skilled tasks, so relevant knowledge in both species is crucial for cooperation to function. First, both species need an initial understanding of the communication and basic actions involved in the interaction. This understanding is probably culturally learnt in all human partners and in dolphins (e.g., Catão & Barbosa, 2018; Simões‐Lopes et al., 2016), while the tendency to initiate cooperation appears to be innate in honeyguides, and is likely further refined by learning (Box 1). In many parts of Africa where honeyguides still attempt to guide humans, human partners have lost the cultural knowledge or interest to engage in the interaction (e.g., Gruber, 2018). Second, human partners vary geographically in traits relevant to the wildlife partner, for example, in the signals used to communicate with honeyguides (Laltaika, 2021; Spottiswoode et al., 2016; van der Wal et al., 2022; Wood et al., 2014) and dolphins (Peterson et al., 2008; Smith et al., 2009; Tun, 2004, 2014), in the tools used to access the resource (Laltaika, 2021; Simões‐Lopes et al., 1998), and in cultural attitudes toward rewarding the wildlife partner (Isack, 1999; Laltaika, 2021; Neil, 2002; Spottiswoode et al., 2016; Wood et al., 2014). In turn, the wildlife partner must learn and behaviorally adapt to such variation (particularly where it affects the interaction's coordination through cues and signals), to make the interspecies knowledge compatible. In theory, this means cooperation is vulnerable to external factors (such as human displacement) that could cause a mismatch in knowledge. Local incompatibility in knowledge could cause less efficient or wholly nonfunctioning cooperation. Nevertheless, most conservation efforts fail to sufficiently consider human and nonhuman animal cultures (Brakes et al., 2021).

Skills required to engage in human–wildlife cooperation could be eroded or permanently lost when younger individuals are unwilling or unable to learn them, and older individuals, who often represent “repositories” of the requisite skills and knowledge, die. In some dolphin populations, certain individuals are recognized by humans to be particularly effective co‐operators (da Rosa et al., 2020; Peterson et al., 2008; Zappes et al., 2011). If these individuals contribute disproportionately to the persistence of the interaction, an assessment of population size may underestimate the vulnerability of the interaction. Moreover, social learning requires repeated exposure to learning opportunities, and if the density or temporal and spatial mobility of either motivated human or wildlife partners critically declines, learning opportunities may be insufficient to maintain the cooperative behavior (Whitehead, 2010). In Torres in Brazil, illegal fishing killed one of two dolphins that sporadically cooperated with fishers (Gonçalves, 2018), highlighting how precarious these interactions can be. In human communities, even where knowledgeable individuals are available as demonstrators, younger generations may not be encouraged, or may be less willing, to learn the required skills, for example, because of increased opportunities for Western education and/or alternative income (Ilha et al., 2020; Isack, 1999; Laltaika, 2021; van der Wal et al., 2022).

### 3.5 Causes of extinction in inactive human–wildlife cooperation and obstacles to restoration

Understanding the reasons for the demise of inactive examples of human–wildlife cooperation can inform our assessment of risks to active cases. The major contributing factor to the extinction of past cases appears to be destructive interference from human outsiders harming local people and/or their wildlife partners, causing permanent cultural change (Figure 2). Cooperation with Indo‐Pacific bottlenose dolphins (*Tursiops aduncus*) in Moreton Bay, Australia, reportedly disappeared because European settlers deliberately killed a dolphin, extinguishing the dolphin population's motivation to cooperate (Neil, 2002). Similarly, European settlers in Australia killed two cooperating orcas at Twofold Bay, causing many orcas to leave the area (Clode, 2002). Moreover, the wholesale slaughter of baleen whales likely caused orcas to move to more productive hunting grounds (Clode, 2002). When European settlers moved to the Great Plains of North America in the 19th century, they killed wolves, ungulates, and displaced or killed Indigenous people, eliminating all components of the human–wolf relationship (Fogg et al., 2015; Pierotti & Fogg, 2017; Standing Bear, 1978). Advances in human technology may also have contributed to cessation of human–wildlife cooperation (e.g., Clode, 2002). Modern methods of catching and killing prey, including rifles, exploding harpoons, motorboats, and larger fishing gear, all reduced the need to cooperate with wolves, dolphins, and orcas, and in many cases the associated noise and risk of injury deters willing cooperative wildlife partners (e.g., Pellegrini et al., 2021).

Once the wildlife partner's specialized behavior or compatible knowledge is lost, restoration is likely to be very difficult. Although wolf (and prey) populations have somewhat recovered, motivated Indigenous peoples are forbidden from hunting in national parks, and wolves fear and avoid humans (Pierotti & Fogg, 2017). If viable wildlife partner populations are still present, it may be feasible to reignite human–dolphin and human–honeyguide cooperation where these disappeared recently (e.g., one or two generations ago), or where active cooperative sites, from which human knowledge may be transmitted, are nearby. Nevertheless, given the challenges of restoring lost cases of human–wildlife cooperation, efforts are better focused on safeguarding active cases.

## 4 HOW CAN HUMAN–WILDLIFE COOPERATION BE SAFEGUARDED FOR FUTURE GENERATIONS?

### 4.1 An ethical, inclusive, and sustainable approach

Safeguarding measures should be based on the needs and interests of the participating human and wildlife communities, and should consider wider ecosystem consequences. Interventions should always follow the highest ethical standards (International Society of Ethnobiology, 2006) and the principle of Free Prior Informed Consent that is central to the United Nations Declaration on the Rights of Indigenous Peoples (UN General Assembly, 2007). Where safeguarding measures are encouraged by external entities, these should be designed in collaboration with local stakeholders through transparent co‐management (Staddon et al., 2021). Any activities to encourage human–wildlife cooperation should also consider the carrying capacity of the environment, although there is no evidence of depletion of fish stocks or bee populations as a direct result of human–wildlife cooperation. Clearly, decisions about whether and how people outside these systems (e.g., researchers, conservationists, governments) should become involved in safeguarding human–wildlife cooperation need to be justified and informed on a case‐by‐case basis (Gavin et al., 2015).

### 4.2 Conserving opportunities for human participation

Where possible, conservation strategies aimed at protecting other aspects of local biodiversity should be permissive and supportive of human engagement in human–wildlife cooperation, and tolerant of the associated human cultural practices (Dowie, 2009). Where Indigenous and local communities have access to protected areas, decision makers should understand and integrate cultural practices associated with human–wildlife cooperation, reducing conflict and confrontation (e.g., with antipoaching scouts in honey‐hunting habitats; van der Wal et al., 2022). To best support local communities in maintaining their income and livelihoods, decision makers need to understand both the economic and cultural value of human–wildlife cooperation (Machado, Daura‐Jorge, et al., 2019). It is also key to consider how certain sustainable development strategies can indirectly threaten human–wildlife cooperation, such as the widespread promotion of beekeeping in many African communities (Illgner et al., 1998), or total bans on tree felling. Raising awareness of human–wildlife cooperation among the implementers of such schemes should reduce chances of inadvertently negative outcomes for human–wildlife cooperation.

Campaigns to raise awareness can also encourage enthusiasm about a given case of human–wildlife cooperation, reinforcing positive attitudes and behaviors toward the interaction, and bolstering motivation to conserve it (Smith et al., 2020). More generally, favorable outsider interest from researchers, conservationists, journalists, artists, and tourists is likely to invigorate communities’ sense of pride in their local cultural and ecological knowledge and practices, affirming that these have special value and are worth protecting. Forms of human–wildlife cooperation also provide powerful examples of successful human–wildlife coexistence that can inspire other conservation partnerships to similarly engage with local wildlife custodians in decision‐making and protected area management.

Human participation in cooperative partnerships can be maintained and protected by targeted incentivization, for example, premium prices paid to people for products they personally obtain from human–wildlife cooperation, where there is external market demand. In Brazil, fishers are typically restricted to selling their catch on‐site, meaning only places that regularly receive tourists can set a premium price for fish caught with the help of dolphins (Machado, Daura‐Jorge, et al., 2019). Moreover, since resources can also be acquired without help of the wildlife partner, the system is vulnerable to counterfeit products. Facilitating a market that implements a certification scheme might benefit human partners and their communities. Tourism may provide alternative opportunities for financial support to the communities involved, as has been demonstrated in Hadza hunter‐gatherer communities in Tanzania (honeyguides) and in some fishing communities in Brazil and Myanmar (dolphins). Income from these sources relies primarily on existing tourist infrastructure and may therefore be unfeasible in more remote or unstable regions.

Efforts to maintain human–wildlife cooperation should guard against possible unintended negative outcomes. Financial incentivization schemes can attract opportunistic people to the system, which can jeopardize the interaction if newcomers (i) cause overexploitation of the environment, (ii) exploit or harm the wildlife partner, and/or (iii) affect the behavioral dynamics of the cooperation, for example, by introducing greater variability in “partner quality” for the wildlife partner. Schemes may need to limit such harmful opportunism by finding ways of targeting incentives to those whose genuine engagement in human–wildlife cooperation is declining under financial pressure.

### 4.3 Conserving opportunities for wildlife participation

Conservation strategies targeted at wildlife partner populations require good understanding of the ecology of the wildlife species. Wildlife populations need a relatively intact habitat without human‐induced risk and mortality, so enforcement of legal protection is required. In southern Brazil, current legislation bans gill and trammel nets near cooperative sites, which, if combined with enforcement operations, could successfully reduce dolphin bycatch (Bezamat et al., 2021). In Myanmar, increased patrolling will help battle illegal fishing, by enforcing the 2018 legislation protecting Irrawaddy dolphins and limiting net deployment at cooperative sites. Better vessel traffic control, stricter policing of (illegal) fisheries, commercial fishing quotas, and consideration of the vulnerability of human–dolphin cooperation in plans for urban development in coastal areas will all be crucial for conserving dolphin populations that cooperate with humans (e.g., Zappes et al., 2011).

### 4.4 Conserving a suitable environment

Effectively protecting the environment that supports human–wildlife cooperation also requires an understanding of the ecology and habitat of prey species. Human–dolphin cooperation requires sufficient fish availability, and attempts to prevent fish stock collapse must be enforced through more sustainable fisheries management (de Abreu‐Mota et al., 2018). In a cautionary example, stricter regulations by Brazilian authorities on industrial‐ and small‐scale mullet fisheries have been largely ineffective for ensuring the maintenance of regional mullet stock (de Abreu‐Mota et al., 2018; Sant'Ana et al., 2017). Lagoons and estuarine areas along the southern Brazilian coastline also require protection, as the mullet's life cycle depends on them. The Irrawaddy River Corridor is being considered a potential World Natural Heritage Site, in part because it supports cooperation between the Irrawaddy dolphin and fishers (UNESCO, 2014). Where deforestation is locally threatening honeybee populations (Laltaika, 2021), protection of forests that provide nesting and foraging opportunities for bees will help ensure the continuation of human–honeyguide cooperation. The wider conservation crises that have devastated fish stocks and forests globally therefore also threaten the only remaining cases of human–wildlife cooperation. Their future depends on effective ecosystem protection, which can be enhanced by the involvement of Indigenous and local communities that support human–wildlife cooperation (Garnett et al., 2018; Reid et al., 2021).

### 4.5 Safeguarding cultural transmission and compatible knowledge

The importance of considering animal culture in biological conservation is now increasingly recognized (Brakes et al., 2019; Carvalho et al., 2022). Cultural transmission of skills required for human–wildlife cooperation is in itself important to safeguard, because it can increase resilience by stabilizing cooperative systems when participants increase or decrease in population size, and can allow their expansion into new areas (Whitehead, 2010). Considering both human and nonhuman animal cultural diversity in conservation can alter best practices in several ways. First, while the majority of conservation practices prioritize genetic diversity and individuals with the highest reproductive potential (e.g., Eizaguirre & Baltazar‐Soares, 2014), strategies informed by cultural diversity may instead favor individuals who can serve as reliable demonstrators (e.g., Brent et al., 2015). Long‐term empirical work is needed to identify these key individuals, and conservation efforts should be aimed at protecting their vital role in the transmission of cultural knowledge (Brakes et al., 2019). For example, in Laguna, the most competent cooperative dolphins have been identified, as well as their home ranges (Cantor et al., 2018). Focusing on protecting those specific areas would better protect these individuals, thereby retaining knowledge on how to cooperate with humans in the population. Second, the role that cultural variation plays in shaping adaptive phenotypes demands a re‐assessment for defining “conservation units.” In addition to genetic variants, conservationists may need to identify “cultural variants” of wildlife populations that require tailored conservation strategies (Brakes et al., 2019). Identifying cultural variants takes time, given it requires demonstrating that behaviors are socially learnt and cannot be explained by ecological and genetic factors. In declining populations, such evidence may only become available after it is too late (Cabin, 2007). Where animal cultural transmission is suspected to play a role in highly vulnerable cases of human–wildlife cooperation, a precautionary approach would be to implement measures to safeguard cultural transmission (Carvalho et al., 2022).

Even when appropriate knowledge exists in both partners, local variants need to be interspecifically compatible for cooperation to function well. This need is especially apparent for signals used to communicate with the partner species, which vary culturally among human populations, and possibly also in the wildlife partner (Laltaika, 2021; Serpa, 2019; Simões‐Lopes et al., 1998; Spottiswoode et al., 2016; van der Wal et al., 2022; Wood et al., 2014). Effective safeguarding strategies must ensure that local cultural knowledge in both partner species persists in the same place at the same time.

### 4.6 Promoting and archiving knowledge

Organizations such as the United Nations Educational, Scientific and Cultural Organization (UNESCO) provide guidance and standards on identifying and safeguarding cultural heritage, for example, by recognizing “Intangible Cultural Heritages” to preserve non‐monumental, living human culture (UNESCO, 2020). Documenting remaining cooperative sites under the umbrella of Intangible Cultural Heritage has the potential to bring economic benefits through tourism, and to organize efforts to work collaboratively with (and attract funding for) motivated local partners interested in research and/or conservation. In Brazil, human–dolphin cooperation is already recognized by domestic legislation as Intangible Cultural Heritage in the state of Rio Grande do Sul (state law 15.546/RS, 2020) and in the municipality of Laguna, Santa Catarina (municipal law 17.084/2017, 2017). Preparations to recognize these and other human–wildlife cooperative sites and forms as Intangible Cultural Heritage are underway. These efforts must consider, first, that data collection efforts and registration of a cooperative site as Intangible Cultural Heritage should respect intellectual property and confidentiality, and retain rich information documenting the context of activities; second, that UNESCO registration can accelerate change in ways that might threaten the partnership, for example, through attracting opportunistic human partners (Deacon et al., 2004).

Archiving diverse information relating to the biological and cultural aspects of human–wildlife cooperation will require engagement and partnerships with local communities, scientists, and conservation practitioners. The resulting outputs should be widely available in all relevant languages. Diverse forms of media can be used to raise public awareness about human–wildlife cooperation, including books, social media, and museum exhibitions. Data and media can be collected by local communities themselves, along with scientists and other members of the public (e.g., *pescacombotos.art.br*). Web platforms including multimedia content can be open‐access or restricted where appropriate. These platforms also permit citizen science data collection (e.g., *Honeyguiding.me*; van der Wal & Spottiswoode, 2020) and open dialogue between the public and people engaged in human–wildlife cooperation (e.g., www.ewatlas.net).

### 4.7 Empirical anthropological and behavioral ecology studies

**FIGURE 3:**
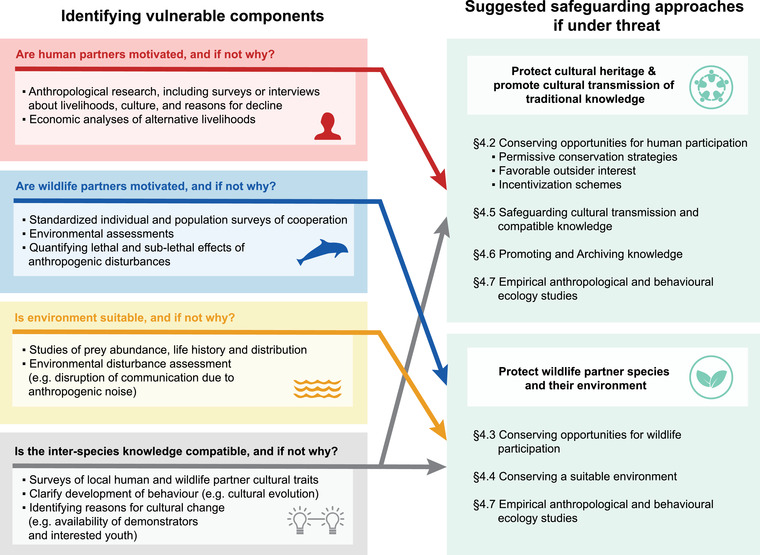
A flow‐chart to help researchers and conservation decision makers identify the weak component(s) of human–wildlife cooperation, with the associated suggested broad safeguarding approaches (numbering refers to sections in main text).

In most cases of human–wildlife cooperation, key information to inform safeguarding policies is lacking. First, we need a good understanding of where human–wildlife cooperation occurs, which involves prioritizing research on cases of human–wildlife cooperation that are poorly documented, unconfirmed, or unknown to science. Second, research into the ecology and evolution of human–wildlife cooperation, and conservation status of the wildlife partner, is urgently needed to examine fitness benefits to both human and nonhuman parties, knowledge transmission pathways, and wider impacts on the local ecosystem. Third, local threats and responses to environmental and cultural change at cooperative sites need to be identified (Figure 1), especially where cooperation has recently declined or become inactive. To do this, we need to involve both researchers and stakeholders. Effective solutions will require insights and methods from the social and biological sciences, such as observational ethnographic research (e.g., Gruber, 2018; Wood et al., 2014) and targeted surveys and semi‐structured interviews (e.g., Fogg et al., 2015; Laltaika, 2021; Machado, Daura‐Jorge, et al., 2019; van der Wal et al., 2022), animal behavior field studies (Cantor et al., 2018; Spottiswoode et al., 2016), and long‐term population monitoring (Bezamat et al., 2018) (Figure 3). Data collection and community engagement protocols must be jointly developed by researchers and local communities (Adams et al., 2014), ensuring best practices for engaging with human participants (Tunón et al., 2016) and wildlife (Sikes et al., 2019).

## 5 CONCLUSIONS AND WIDER IMPLICATIONS

While mitigating human–wildlife conflict has received extensive attention by conservationists, the cultural and ecological value and vulnerability of human–wildlife cooperation have been neglected. The diverse and complex nature of these systems poses unique challenges for conservation. Safeguarding strategies must lie at the interface between animal culture conservation (Brakes et al., 2019) and human cultural heritage conservation (Bridgewater & Rotherham, 2019). They must mitigate threats while considering the needs of the human and nonhuman parties, availability of suitable environments, and persistence of compatible interspecies knowledge. Research and conservation efforts need to be logistically and politically feasible, ethically engaged, locally appropriate, and highly collaborative. The biological and cultural diversity between and within cases of human–wildlife cooperation requires tailored safeguarding plans. Further work is also required to identify new and locally varying cases of human–wildlife cooperation. Scientists, conservationists, and local communities should collaborate to identify the specific threats that cases of human–wildlife cooperation face and how to mitigate these, to create public awareness, and to document the irreplaceable aspects of cultural heritage they represent.

Although the combination of conservation challenges faced by cases of human–wildlife cooperation is unique, the issues and strategies outlined above are consistent with recent advances in conservation. In particular, recent work has highlighted the need to consider animal culture (Brakes et al., 2019; Carvalho et al., 2022), and the interplay between local human and animal cultures (referred to as the “biocultural paradigm” in biological anthropology; Bridgewater & Rotherham, 2019; Gavin et al., 2015) in conservation decision‐making. Such interspecies cultural interactions are not restricted to human–wildlife cooperation: human cultural variation is so pervasive that it is likely that wherever animal culture exists, it will also interact with local human culture. Conservation consideration of the species’ cultures and their interactions could, for example, improve the identification of conservation units including cultural keystone species, inform the tailoring of strategies to the needs of specific populations, and raise public engagement in conservation through the promotion of flagship positive human–wildlife interactions. Moreover, our review stresses some of the harmful consequences that “fortress conservation” models can have, and adds to work emphasizing that the removal of humans from a habitat must not be a default goal of conservation strategies (Jones, 2007). Broadly, our review highlights that efforts to maintain, restore, enhance, and archive biological and cultural diversity, ecosystem services, and ecosystem function should carefully consider the unique, varied, and impactful interactions between local human and animal cultures.

## AUTHOR CONTRIBUTIONS

J.E.M.vd.W., C.N.S., N.T.U., M.C., F.G.D.‐J., and D.L.C. made the largest contribution to this publication. J.E.M.vd.W., D.L.C., C.N.S., and N.T.U. conceptualized the study, with input from all co‐authors. J.E.M.vd.W. and D.L.C. led the literature search, with input from all co‐authors. J.E.M.vd.W. led the writing of the manuscript, with significant support from D.L.C., C.N.S., M.C., and F.G.D.‐J. and contributions from all co‐authors. M.C. prepared the figures with input from J.E.M.vd.W., D.L.C., and C.N.S. All authors approved the final version for submission.

## CONFLICT OF INTEREST

The authors declare no conflict of interest.

## Supporting information

Table S1: Documented active and inactive cases of human‐wildlife cooperation, either from published literature or through personal observation by authors on this paper.
Table S2 Documented active and inactive cases of human‐wildlife interactions that are potential mutualistic and/or cooperative, or that are mutualistic but not cooperative. Based on published literature or through personal observation by authors on this paper.
Table S3: Causes of decline and loss for active and inactive forms of human‐wildlife cooperation, respectively. Text is reproduced from Fig. 2 in main text, here with associated references.
