# Supplementary material for: Safeguarding human–wildlife cooperation
Source: Conserv Lett. 2022 Jun 9;15(4):e12886. doi: 10.1111/conl.12886 (PMC9540276; doi:10.1111/conl.12886)
Supplement: Supplementary file 1 — Table S1: Documented active and inactive cases of human‐wildlife cooperation, either from published literature or through personal observation by authors on this paper. Table S2 Documented active and inactive cases of human‐wildlife interactions that are potential mutualistic and/or cooperative, or that are mutualistic but not cooperative. Based on published literature or through personal observation by authors on this paper. Table S3: Causes of decline and loss for active and inactive forms of human‐wildlife cooperation, respectively. Text is reproduced from Fig. 2 in main text, here with associated references. [file CONL-15-e12886-s001.pdf]

Supplementary Material

Table S1: Documented active and inactive cases of human-wildlife cooperation, either from published literature or through personal observation by authors on this paper.

Human-wildlife cooperation	Non-human animal species	Human cultural group	Location	Prey species	Status and period	References
Human-dolphin	Lahille's bottlenose dolphin (*Tursiops truncatus gephyreus* or *Tursiops gephyreus*)*	Artisanal Brazilian net-casting fishers	Araranguá, Santa Catarina, southern Brazil	Mainly Mugilidae	Inactive, ~ 1900–1991	(Simões-Lopes, 1991)
Human-dolphin	Lahille's bottlenose dolphin (*Tursiops truncatus gephyreus* or *Tursiops gephyreus*)*	Artisanal Brazilian net-casting fishers	Laguna, Santa Catarina, southern Brazil	Mainly Mugilidae	Active (ca. 16 cooperative dolphins), ca. 1890–current day	(Bezamat et al., 2018, 2019; da Rosa et al., 2020; Daura-Jorge et al., 2012, 2013; Machado et al., 2019a; b; Pellegrini et al., 2021; Peterson et al., 2008; Pryor & Lindbergh, 1990; Romeu et al., 2017; Simões-Lopes, 1991; Simões-Lopes et al., 1998, 2016)
Human-dolphin	Lahille's bottlenose dolphin (*Tursiops truncatus gephyreus* or *Tursiops gephyreus*)*	Artisanal Brazilian net-casting fishers	Rio Grande, Rio Grande do Sul, Southern Brazil	Mainly Mugilidae	Inactive ~ 1900–1991	(Simões-Lopes, 1991)
Human-dolphin	Lahille's bottlenose dolphin (*Tursiops truncatus gephyreus* or *Tursiops gephyreus*)*	Artisanal Brazilian net-casting fishers	Torres river, Rio Grande do Sul, southern Brazil	Mainly Mugilidae	Active (1–2 cooperative dolphins), much reduced, ca. 1900–current day	(Gonçalves, 2018; Simões-Lopes, 1991)
Human-dolphin	Lahille's bottlenose dolphin (*Tursiops truncatus gephyreus* or *Tursiops gephyreus*)*	Artisanal Brazilian net-casting fishers	Tramandaí Inlet, Rio Grande do Sul, southern Brazil	Mainly Mugilidae	Active (ca. 12 cooperative dolphins), ca. 1900–current day.	(Afonso, 2015; Camargo et al., 2020; Ilha et al., 2018, 2020; Santos et al., 2018; Serpa, 2019; Silva et al., 2021; Simões-Lopes, 1991; Zappes et al., 2011, I.B.M., unpubl. data)
Human-dolphin	Irrawaddy dolphin (*Orcaella brevirostris*)	Artisanal Brazilian net-casting fishers	Ayeyarwady river, Sagaing and Mandalay Regions, Myanmar	Mainly Cyprinidae	Active (ca. 8 cooperative dolphins), at least 1878– current day	(Anderson, 1878; Busnel, 1973; Smith et al., 2009; Thein, 1977; Tun, 2004, 2005, 2014)

Human-wildlife cooperation	Non-human animal species	Human cultural group	Location	Prey species	Status and period	References
Human-dolphin	Indo-Pacific bottlenose dolphin (*Tursiops aduncus)*	Bunjalung Aboriginal Australians using spears and hand nets	Eastern Australia	Mugilidae and Pomatomidae	Inactive	(Clode, 2002; Fairholme, 1856; Neil, 2002; Robinson, 1965)
Human-orca	Orca (*Orcinus orca*)	Yuin Aboriginal Australians, together with European settlers	Eastern Australia	Baleen whales	Inactive	(Clode, 2002; Dakin, 1938; Mead, 1961; Neil, 2002)
Human-orca	Orca (*Orcinus orca*)	Chukchi, Siberian Yupik	Chukotka, Russia	Walruses (*Obobenus rosmarus*), true seal species (Phocidae), grey whales (*Eschrichtius robustus*)	Inactive	(Bogoras, 1907; Holzlehner, 2015)
Human-honeyguide	Greater honeyguide (*Indicator indicator*)	Diverse backgrounds	Central Cameroon	Honeybee (*Apis mellifera*)	Inactive	(Gruber, 2018; Gruber & Sanda, 2019)
Human-honeyguide	Greater honeyguide (*Indicator indicator*)	Unknown	Central Mozambique	Honeybee (*Apis mellifera*)	Active	(dos Santos, 1609)
Human-honeyguide	Greater honeyguide (*Indicator indicator*)	Unknown	Congo Basin	Honeybee (*Apis mellifera*)	Likely inactive	(Chapin, 1939; Friedmann, 1955; Merolla da Sorrento, 1744)
Human-honeyguide	Greater honeyguide (*Indicator indicator*)	Xhosa	Eastern Cape, South Africa	Honeybee (*Apis mellifera*)	Inactive	(Friedmann, 1955; Skead, 1951)
Human-honeyguide	Greater honeyguide (*Indicator indicator*)	Swazi	Kingdom of Eswatini	Honeybee (*Apis mellifera*)	Active	(G.S.D, unpubl. data)
Human-honeyguide	Greater honeyguide (*Indicator indicator*)	Awer	Lamu County, Kenya	Honeybee (*Apis mellifera*)	Active	(van der Wal et al., 2022)

Human-wildlife cooperation	Non-human animal species	Human cultural group	Location	Prey species	Status and period	References
Human-honeyguide	Greater honeyguide (*Indicator indicator*)	Yao	Niassa Special Reserve, Northern Mozambique	Honeybee (*Apis mellifera*), meliponine stingless bee species	Active	(Spottiswoode et al., 2016)
Human-honeyguide	Greater honeyguide (*Indicator indicator*)	Boran	Northern Kenya	Honeybee (*Apis mellifera*)	Active,	(Isack, 1987, 1999; Isack & Reyer, 1989)
Human-honeyguide	Greater honeyguide (*Indicator indicator*)	Hadzabe	Northern Tanzania	Honeybee (*Apis mellifera*), rarely meliponine stingless bee species	Active	(Laltaika, 2021; Marlowe et al., 2014; Wood et al., 2014)
Human-honeyguide	Greater honeyguide (*Indicator indicator*)	Ndorobo	Northern Tanzania	Honeybee (*Apis mellifera*)	Active	(Laltaika, 2021; Queeny, 1952)
Human-honeyguide	Greater honeyguide (*Indicator indicator*)	Sonjo	Northern Tanzania	Honeybee (*Apis mellifera*)	Active	(Laltaika, 2021)
Human-honeyguide	Greater honeyguide (*Indicator indicator*)	Maasai	Northern Tanzania	Honeybee (*Apis mellifera*)	Active	(Laltaika, 2021)
Human-honeyguide	Greater honeyguide (*Indicator indicator*)	Unknown	Present-day Ethiopia	Honeybee (*Apis mellifera*)	Unknown	(Friedmann, 1955; Lobo, 1789)
Human-honeyguide	Greater honeyguide (*Indicator indicator*)	Unknown	Present-day Guinea	Honeybee (*Apis mellifera*)	Likely inactive	(Friedmann, 1955; Ludolphus, 1682)
Human-honeyguide	Greater honeyguide (*Indicator indicator*)	Waata	Tsavo, southern Kenya	Honeybee (*Apis mellifera*)	Unknown	(Ville, 1995)
Human-honeyguide	Greater honeyguide (*Indicator indicator*)	Khoe-Sān	Western Cape, South Africa	Honeybee (*Apis mellifera*)	Inactive	(Sparrman, 1777)
Human-wolf	Wolf (*Canis lupus*)	Indigenous Americans	North America	Various ungulates, including bison (*Bison bison*) and elk (*Cervus Canadensis*)	Inactive, possibly active in limited areas	(Barsh & Marlor, 2003; Fogg et al., 2015; Marshall, 1995; Pierotti & Fogg, 2017; Shipman, 2015)

* There is an ongoing taxonomic debate on Lahille's bottlenose dolphin should be considered a species (*Tursiops gephyreus*) or a subspecies (*Tursiops truncatus gephyreus*) (e.g., Wang et al., 2021; Wickert et al., 2016)

Table S2 Documented active and inactive cases of human-wildlife interactions that are potential mutualistic and/or cooperative, or that are mutualistic but not cooperative. Based on published literature or through personal observation by authors on this paper.

Assessment	Human-wildlife interaction	Wild animal species	Human cultural group	Location	Prey species	Status and period	Notes	References
Mutualism; no cooperation	Human-dolphin	Guiana dolphin (*Sotalia guianensis*)	Artisanal Brazilian fishers using unsupervised stake nets	Cananéia, south-eastern Brazil	Mainly Mugilidae	Active, 1982–current day	The use of stake-nets indicates no real-time coordination	(Louzada, 2013; Monteiro-Filho, 1995; Monteiro-Filho et al., 2018)
Potential mutualism/parasitism	Human-dolphin	Guiana dolphin (*Sotalia guianensis*)	Brazilian net-casting fishers	Guaratuba southern Brazil	Unknown	Inactive, unknown start date	Not enough details to indicate dolphins benefit	(Monteiro-Filho et al., 1999)
Potential mutualism/parasitism	Human-dolphin	Amazon river dolphin (*Inia geoffrensis*)	Artisanal Brazilian fishers	Manaus	Unknown	Inactive, unknown start date–ca. 1954	Not enough details to indicate dolphins benefit	(Busnel, 1973; Lamb, 1954)
Potential mutualism/parasitism	Human-dolphin	Indo-Pacific humpback dolphin (*Sousa chinensis*)	Ashtamudi artisanal fishers	Ashtamudi, south-western India	Mainly Mugilidae	Active, unknown start date	Not enough details to indicate dolphins benefit	(Kumar et al., 2012)
Mutualism; no cooperation	Human-dolphin	Irrawaddy dolphin (*Orcaella brevirostris*)	Chilika artisanal fishers using unsupervised stake nets	Chilika, eastern India	Mainly Mugilidae	Active, unknown start date	The use of stake-nets indicates no real-time coordination	(D'Lima et al., 2014)
Potential mutualism/parasitism	Human-dolphin	South Asian river dolphin (*Platanista gangetica gangetica*)	Artisanal fishers	Sundarbans, Bangladesh	Mugilidae and other small fish and shrimp species	Active, unknown start date	It seems unlikely that dolphins benefit, probably not mutualistic	(Deb, 2015)
Mutualism; potential cooperation	Human-dolphin	Common bottlenose dolphin (*Tursiops truncatus*)	Imragen fishers using spears and hand nets	El-Memghar, Mauritania	Mainly Mugilidae	Potential active	Not enough details to indicate there is coordination	(Busnel, 1973; Campredon & Cuq, 2001)

Assessment	Human-wildlife interaction	Wild animal species	Human cultural group	Location	Prey species	Status and period	Notes	References
Mutualism; potential cooperation	Human-dolphin	Atlantic humpback dolphin (*Sousa teuszii*)	Imragen fishers using spears and hand nets	El-Memghar, Mauritania	Mainly Mugilidae	Unknown	Not enough details to indicate there is coordination	(Busnel, 1973)
Mutualism; potential cooperation	Human-dolphin	Unknown dolphin species	Unknown	Iasos gulf, present-day Turkey	Unknown	Inactive, unknown start and end dates	Not enough details to indicate there is coordination	(Orams, 1997; Ridgway, 1970; Stebbins, 1929; Turgut, 2010)
Mutualism; potential cooperation	Human-dolphin	Unknown dolphin species	Artisanal fishers	Montpellier, present-day France	Mainly Mugilidae	Inactive, unknown start and end dates	Not enough details to indicate there is coordination	(Pliny the Elder. A.D. 23-79, 1940)
Mutualism; potential cooperation	Human-dolphin	Unknown dolphin species	Unknown	Palomos gulf, present-day Spain	Unknown	Inactive, unknown start and end dates	Not enough details to indicate there is coordination	(Orams, 1997; Ridgway, 1970; Stebbins, 1929; Turgut, 2010)
Potential mutualism	Human-orca	Orca (*Orcinus orca*)	Gilyak, Nivkhy	Kamchatka, Russia	Walruses (*Obobenus rosmarus*), seal species (Phocidae), grey whales (*Eschrichtius robustus*)	Inactive	Not enough details to indicate orcas benefit	(Jochelson, 1908; Shternberg, 1933)
Potential mutualism	Human-orca	Orca (*Orcinus orca*)	Yamana, Selknam	Cape Horn, Patagonia	Baleen whales (Mysticeti)	Inactive	Not enough details to indicate orcas benefit	(Bogoras, 1907; Chapman, 1997, 2010)
Mutualism; potential cooperation	Human-orca	Orca (*Orcinus orca*)	Nunavut Inuit	Western Hudson Bay (Kangiqsualuk ilua), central Canada	Narwhal, Beluga, Bowhead (*Balaena mysticetus*), seals	Inactive	Not enough details to indicate there is coordination	(Westdal et al., 2017)
Potential mutualism	Human-honeyguide	Lesser honeyguide (*Indicator minor*)	Baka	Congo Basin	Honeybee (*Apis mellifera*)	Unknown	Not enough details available to confirm mutualism	(Brisson, 2010; Dounias, 2018)

Assessment	Human-wildlife interaction	Wild animal species	Human cultural group	Location	Prey species	Status and period	Notes	References
Potential mutualism	Human-honeyguide	Dwarf honeyguide (*Indicator pumilio*)	Batwa	South-western Uganda	Stingless bee species (probably meliponine)	Unknown	Not enough details available to confirm mutualism	(Kajobe & Roubik, 2007)
Potential mutualism	Human-honeyguide	Scaly-throated honeyguide (*Indicator variegatus*)	Unknown	Several places in South and East Africa	Honeybee (*Apis mellifera*)	Unknown	Not enough details available to confirm mutualism	(Friedmann, 1955; Ivy, 1901)
Potential mutualism	Human-wolf	Wolf (*Canis lupus*)	Diverse backgrounds	Several places in Europe	Ungulates	Inactive, c.a. 32,000 years ago (late Pleistocene)	Not enough details available to confirm mutualism	(Crockford & Kuzmin, 2012; Germonpré et al., 2009; Ovodov et al., 2011)
Potential mutualism	Human-wolf	Wolf (*Canis lupus*)	Ainu	Hokkaido, Japan	Sika deer (*Cervus nippon*)	Inactive, 18th century	Not enough details available to confirm mutualism	(Walker, 2005)
Mutualism; no cooperation	Human-wolf	Wolf (*Canis lupus*)	Koyukon	Alaska	Ungulates	Potentially active	Possibly a mutualism, but not a cooperative one	(Nelson, 1983)
Potential mutualism	Human-corvid	Common ravens (*Corvus corax*)	Diverse backgrounds	North America, Europe and Arctic	Ungulates	Potentially active	Not enough details available to confirm mutualism	(Freuchen & Solomonsen, 1958; Heinrich, 1999)
Potential mutualism	Human-corvid	New Caledonian crows (*Corvus moneduloides*)	Kanak	New Caledonia	Longhorn beetle larvae (*Agrianome fairmairei*)	Potentially active	Not enough details available to confirm mutualism	(N.T.U., unpubl. data)

Table S3: Causes of decline and loss for active and inactive forms of human-wildlife cooperation, respectively. Text is reproduced from Fig. 2 in main text, here with associated references.

	**Human-dolphin cooperation**	**Human-honeyguide cooperation**	**Human-orca cooperation**	**Human-wolf cooperation**
**Human partner**	Alternative fisheries or sources of income, urban encroachment	Changing livelihoods (incl. beekeeping), other sugar sources, displacement from national parks	Displacement by settlers, changing livelihoods	Displacement and extermination by settlers
	(Campredon & Cuq, 2001; D'Lima et al., 2014; Ilha et al., 2020; Machado et al., 2019b; Peterson et al., 2008; Santos-Silva et al., 2022; Smith et al., 2009; Tun, 2004, 2014; Zappes et al., 2011)	*(Dean et al., 1990; Gruber, 2018; Isack, 1999; Laltaika, 2021; van der Wal et al., 2022)*	*(Clode, 2002; Neil, 2002)*	*(Barsh & Marlor, 2003; Fogg et al., 2015; Marshall, 1995; Pierotti & Fogg, 2017; Standing Bear, 1978)*
**Wildlife partner**	Human-induced risk and mortality (bycatch, habitat degradation or loss, noise, pollution)	Potentially deforestation affecting certain host species	Injury or killing of orcas by humans	Hunting of wolves by settlers
	(Agrelo et al., 2019; Bezamat et al., 2021; Camargo et al., 2020; Campredon & Cuq, 2001; Daura-Jorge et al., 2013; Pellegrini et al., 2021; Righetti et al., 2019; Smith et al., 2009; Tun, 2004, 2014; Zappes et al., 2011)	*(C.N.S., unpubl. data)*	*(Clode, 2002; Neil, 2002)*	*(Fogg et al., 2015; Standing Bear, 1978)*
**Suitable environment**	Industrial overfishing, pollution	Droughts affecting bees, deforestation near urban areas	Hunting of whales and other prey	Extermination of ungulates by settlers
	(Agrelo et al., 2019; Camargo et al., 2020; de Abreu-Mota et al., 2018; Pellegrini et al., 2021; Righetti et al., 2019; Santos et al., 2018; Tun, 2004, 2014; Zappes et al., 2011)	*(Gruber, 2018; Gruber & Sanda, 2019; Laltaika, 2021; van der Wal et al., 2022)*	*(Clode, 2002)*	*(Fogg et al., 2015; Standing Bear, 1978)*
**Compatible inter-species knowledge**	Fewer interested youth, loss of skilled demonstrators in both species	Fewer interested youth, loss of skilled demonstrators in both species	Potential skilled demonstrators killed by outsiders	Fear of humans in wolves, loss of opportunity for humans to learn skills
	(Catão & Barbosa, 2018; da Rosa et al., 2020)	*(Isack, 1999; Laltaika, 2021; van der Wal et al., 2022)*	*(Clode, 2002)*	*(Pierotti & Fogg, 2017)*

References Supplementary Material

Afonso, G. S. (2015). *Influência das atividades antrópicas em uma população de botos (Tursiops Gervais, 1855) residente do Litoral Norte do Rio Grande do Sul, Brasil*. Instituto de Biociências; Universidade Federal do Rio Grande do Sul.

Agrelo, M., Daura-Jorge, F. G., Bezamat, C., Silveira, T. C. L., Volkmer de Castilho, P., Rodrigues Pires, J. S., & Simões-Lopes, P. C. (2019). Spatial behavioural response of coastal bottlenose dolphins to habitat disturbance in southern Brazil. *Aquatic Conservation: Marine and Freshwater Ecosystems*, *29*(11), 1949–1958. https://doi.org/10.1002/aqc.3188

Anderson, J. (1878). *Anatomical and Zoological Researches: Comprising an Account of the Zoological Results of the Two Expeditions to Western Yunnan in 1868 and 1875; and A Monograph of the Two Cetacean Genera, Platanista and Orcella[sic].* Bernard Quaritch.

Barsh, R. L., & Marlor, C. P. (2003). Driving bison and Blackfoot science. *Human Ecology*, *31*, 571–593.

Bezamat, C., Castilho, P. V., Simões-Lopes, P. C., Ingram, S. N., & Daura-Jorge, F. G. (2019). Reproductive parameters and factors influencing calf survival of bottlenose dolphins that engage in a unique foraging cooperation with fishermen. *Marine Biology*, *167*(1), 5.

Bezamat, C., Hammond, P. S., Castilho, P. V., Simões-Lopes, P. C., & Daura-Jorge, F. G. (2021). Dolphin population specialized in foraging with artisinal fishers requires zero-bycatch management to persist. *Aquatic Conservation: Marine and Freshwater Ecosystems*, 1–13.

Bezamat, C., Simões-Lopes, P. C., Castilho, P. V., & Daura-Jorge, F. G. (2018). The influence of cooperative foraging with fishermen on the dynamics of a bottlenose dolphin population. *Marine Mammal Science*, *35*(3), 825–842. https://doi.org/10.1111/mms.12565

Bogoras, W. (1907). *Part II. The Chukchee: Religion. Memoirs of the American Museum of Natural History, Vol XI.* G.E. Stechert & Co.

Brisson, R. (2010). *Petit dictionnaire baka-français*. Editions L'Harmattan.

Busnel, R. G. (1973). Symbiotic relationship between man and dolphins. *Transactions of the New York Academy of Sciences*, *2*, 112–131.

Camargo, Y. R., Dal Forno, M., Dorneles, D. R., Frainer, G., Ilha, E. B., Rigon, C. T., Santos, B., Santos, M. L., Serpa, N., Simas, T. P., Carlos, C. J., & Moreno, I. B. (2020). Diagnóstico ambiental do estuário do rio Tramandaí, litoral norte do Rio Grande do Sul, Brasil. *Revista CEPSUL - Biodiversidade e Conservação Marinha*, *9*, e2020002. https://doi.org/10.37002/revistacepsul.vol9.1625e2020002

Campredon, P., & Cuq, F. (2001). Artisanal fishing and coastal conservation in West Africa. *Journal of Coastal Conservation*, *7*(1), 91–100. https://doi.org/10.1007/BF02742471

Catão, B., & Barbosa, G. C. (2018). "Good dolphins", fishes and fishermen: about the conjoint fishing in Laguna (Santa Catarina, Brazil). *Revista Do Instituto de Estudos Brasileiros*, *69*, 205–225.

Chapin, J. P. (1939). The birds of the Belgian Congo. *Bulletin of the American Museum of Natural History*, 550.

Chapman, A. (1997). The Great Ceremonies of the Selk'nam and the Yamana: A Comparative Analysis. In C. McEwan (Ed.), *Patagonia: natural history, prehistory and ethnography at the uttermost end of the earth*. Princeton University Press.

Chapman, A. (2010). *European Encounters with the Yamana People of Cape Horn, Before and After Darwin*. Cambridge University Press.

Clode, D. (2002). *Killers in Eden: The True Story of Killer Whales and Their Remarkable Partnership with the Whalers of Twofold Bay*. Allen & Unwin.

Crockford, S. J., & Kuzmin, Y. V. (2012). Comments on Germonpré et al., Journal of Archaeological Science 36, 2009 "Fossil dogs and wolves from Palaeolithic sites in Belgium, the Ukraine and Russia: Osteometry, ancient DNA and stable isotopes", and Germonpre, Lazkickova-Galetova, and Germonpre, L. *Journal of Archaeological Science*, *39*(8), 2797–2801. https://doi.org/10.1016/j.jas.2012.04.033

D'Lima, C., Marsh, H., Hamann, M., Sinha, A., & Arthur, R. (2014). Positive interactions between Irrawaddy dolphins and artisanal fishers in the Chilika Lagoon of Eastern India are driven by ecology, socioeconomics, and culture. *Ambio*, *43*(5), 614–624. https://doi.org/10.1007/s13280-

013-0440-4

da Rosa, D. S. X., Hanazaki, N., Cantor, M., Simões-Lopes, P. C., & Daura-Jorge, F. G. (2020). The ability of artisanal fishers to recognize the dolphins they cooperate with. *Journal of Ethnobiology and Ethnomedicine*, *16*(1), 1–11. https://doi.org/10.1186/s13002-020-00383-3

Dakin, W. J. (1938). *Whalemen Adventurers: The Story of Whaling in Australian Waters and Other Southern Seas Related Thereto, from the Days of Sails to Modern Times.* Angus & Robertson.

Daura-Jorge, F. G., Cantor, M., Ingram, S. N., Lusseau, D., & Simões-Lopes, P. C. (2012). The structure of a bottlenose dolphin society is coupled to a unique foraging cooperation with artisanal fishermen. *Biology Letters*, *8*(5), 702–705. https://doi.org/10.1098/rsbl.2012.0174

Daura-Jorge, F. G., Ingram, S. N., & Simões-Lopes, P. C. (2013). Seasonal abundance and adult survival of bottlenose dolphins (Tursiops truncatus) in a community that cooperatively forages with fishermen in southern Brazil. *Marine Mammal Science*, *29*(2), 293–311. https://doi.org/10.1111/j.1748-7692.2012.00571.x

de Abreu-Mota, M. A., Medeiros, R. P., & Noernberg, M. A. (2018). Resilience thinking applied to fisheries management: perspectives for the mullet fishery in Southern-Southeastern Brazil. *Regional Environmental Change*, *18*(7), 2047–2058. https://doi.org/10.1007/s10113-018-1323-9

Dean, W. R. J., Siegfried, W. R., & MacDonald, I. A. W. (1990). The fallacy, fact, and fate of guiding behavior in the greater honeyguide. *Conservation Biology*, *4*(1), 99–101. https://doi.org/10.1111/j.1523-1739.1990.tb00272.x

Deb, A. K. (2015). "Something sacred, something secret": traditional ecological knowledge of the artisanal coastal fishers of Bangladesh. *Journal of Ethnobiology*, *35*(3), 536–565. https://doi.org/10.2993/etbi-35-03-536-565.1

dos Santos, J. (1609). *Ethiopia Oriental (Convento de S. Domingo de Évora, Évora)*.

Dounias, E. (2018). Cooperating with the wild: past and present auxiliary animals assisting humans in their foraging activities. In C. Stépanoff & J. D. Vigne (Eds.), *Hybrid communities. Biosocial approaches to domestication and other trans-species relationships.* (pp. 197–220). Routledge.

Fairholme, J. K. E. (1856). The blacks of Moreton bay and the porpoises. *Zoological Society of London, Proceedings*, *11*(497–498).

Fogg, B. R., Howe, N., & Pierotti, R. (2015). Relationships between indigenous American peoples and wolves 1: wolves as teachers and guides. *Journal of Ethnobiology*, *35*(2), 262–285. https://doi.org/10.2993/etbi-35-02-262-285.1

Freuchen, P., & Solomonsen, F. (1958). *The Arctic Year*. Jonathan Cape.

Friedmann, H. (1955). The honey-guides. *Bulletin of the United States National Museum*, *208*, 1–292. https://doi.org/10.5479/si.03629236.208.1

Germonpré, M., Sablin, M., & Stevens, R. (2009). Fossil dogs and wolves from Paleolithic sites in Belgium, the Ukraine and Russia: osteometry, ancient DNA and stable Isotopes. *Journal of Archaeological Science*, *36*, 473–490.

Gonçalves, Y. C. (2018). A visão dos pescadores artesanais sobre a pesca cooperativa e a importância dos Botos, *tursiops truncatus* (MONTAGU, 1821), em dois estuários do sul do Brasil. BSc thesis. In *Universidade Federal do Rio Grande do Sul*.

Gruber, M. (2018). Hunters and guides: multispecies encounters between humans, honeyguide birds and honeybees. *African Study Monographs*, *39*(4), 169–187. https://doi.org/10.14989/236670

Gruber, M., & Sanda, M. (2019). *Honey Hunting and Beekeeping in Adamaoua (Cameroon)*. Rüdiger Köppe Verlag.

Heinrich, B. (1999). *Mind of the Raven*. Harper Collins.

Holzlehner, T. (2015). Werewolves of the Sea: Human-Killer Whale (*orcinus orca*) relationship in the North Pacific. *XI. Conference on Hunting and Gathering Societies, September 7-11, Vienna, Austria*.

Ilha, E. B., Rigon, C. T., Dorneles, D. R., De Camargo, Y. R., Kindel, E. A. I., & Moreno, I. B. (2020). Pescadores e botos: histórias de uma conexão em rede. *Ambiente & Educação*, *25*(2), 512–535. https://doi.org/10.14295/ambeduc.v25i2.8536

Ilha, E. B., Serpa, N. B., Heissler, V. L., Dorneles, D. R., Camargo, Y. R. De, Rigon, C. T., Gass, C. M., Calabrezi, R., Aita, E., Kindel, I., & Moreno, I. B. (2018). *Guia de apoio pedagógico para educadores:*

interação entre pescadores, botos e tainhas: aprendizados sobre cooperação, tradição e cultura. UFRGS.

Isack, H. A. (1987). *The biology of the greater honeyguide Indicator indicator, with emphasis on the guiding behaviour*. DPhil thesis, University of Oxford.

Isack, H. A. (1999). The role of culture, traditions and local knowledge in co-operative honey-hunting between man and honeyguide: a case study of Boran community of northern Kenya. In N. J. Adams & R. H. Slotow (Eds.), *Johannesburg: BirdLife South Africa: Vol. Int. Ornit* (Issue eds Adams; N.J. & Slotow; R.H., pp. 1351–1357). BirdLife South Africa. https://www.internationalornithology.org/PROCEEDINGS_Durban/Symposium/S23/S23.4.htm

Isack, H. A., & Reyer, H. U. (1989). Honeyguides and honey gatherers: interspecific communication in a symbiotic relationship. *Science*, *243*(4896), 1343–1346. https://doi.org/10.1126/science.243.4896.1343

Ivy, R. H. (1901). Notes on the nesting and other habits of some South African birds. *Ibis*, *8*(1), 18–28.

Jochelson, W. (1908). *The Koryak. The Jesup North Pacific Expedition Vol. VI.* E.J. Brill.

Kajobe, R., & Roubik, D. (2007). Honey-making bee colony abundance and predation by apes and humans in a Uganda forest reserve. *Biotropica*, *38*(2), 210–218.

Kumar, A. B., Smrithy, R., & Sathasivam, K. (2012). Dolphin-assisted cast net fishery in the Ashtamudi Estuary, south-west coast of India. *Indian J. Fish.*, *59*(3), 143–148.

Laltaika, E. A. (2021). *Understanding the mutualistic interaction between greater honeyguides and four co-existing human cultures in northern Tanzania*. MSc thesis, University of Cape Town.

Lamb, F. F. (1954). The fisherman's porpoise. *Natural History*, *635*, 231–234.

Lobo, J. (1789). *A voyage to Abyssinia*.

Louzada, C. N. (2013). How do Guiana dolphin *Sotalia guianensis*, from the Cananéia estuary in State of São Paulo, use cerco-fixo fish traps in their fishing activities? *Revista de Etologia*, *12*, 18–24.

Ludolphus, J. (1682). *A new history of Ethiopia*.

Machado, A. M. S., Cantor, M., Costa, A. P. B., Righetti, B. P. H., Bezamat, C., Valle-Pereira, J. V. S., Simões-Lopes, P. C., Castilho, P. V., & Daura-Jorge, F. G. (2019a). Homophily around specialized foraging underlies dolphin social preferences. *Biology Letters*, *15*(4), 5–9. https://doi.org/10.1098/rsbl.2018.0909

Machado, A. M. S., Daura-Jorge, F. G., Herbst, D. F., Simões-Lopes, P. C., Ingram, S. N., Castilho, P. V., & Peroni, N. (2019b). Artisanal fishers' perceptions of the ecosystem services derived from a dolphin-human cooperative fishing interaction in southern Brazil. *Ocean and Coastal Management*, *173*, 148–156. https://doi.org/10.1016/j.ocecoaman.2019.03.003

Marlowe, F. W., Berbesque, J. C., Wood, B. M., Crittenden, A., Porter, C., & Mabulla, A. (2014). Honey, Hadza, hunter-gatherers, and human evolution. *Journal of Human Evolution*, *71*, 119–128. https://doi.org/10.1016/j.jhevol.2014.03.006

Marshall, J. (1995). *On behalf of the wolf and the first peoples*. Red Crane.

Mead, T. (1961). *Killers of Eden: The Story of the Killer Whales of Twofold Bay*. Angus and Robertson.

Merolla da Sorrento, F. J. (1744). A voyage to Congo. In A. Churchill (Ed.), *A collection of voyages and travels* (pp. 521–616).

Monteiro-Filho, E. L. A. (1995). Pesca interativa entre o golfinho *Sotalia fluviatilis guianensis* e a comunidade pesqueira da região de Cananéia. *Boletim Do Instituto de Pesca*, *222*, 15–23.

Monteiro-Filho, E. L. A., Bonin, C. A., & Rautenberg, M. (1999). Interações interespecíficas dos mamíferos marinhs da região da Baia de Guaratuba, litoral sul do Estdo do Paraná. *Biotemas*, *12*, 119–132.

Monteiro-Filho, E. L. A., Deconto, L. S., Louzada, C. N., Wanderley, R. P., Godoy, D. F., & Medeiros, E. (2018). Long-term monitoring of dolphins in a large estuarine system of southeastern Brazil. In *Advances in Marine Vertebrate Research in Latin America* (pp. 15–17). Springer International Publishing.

Neil, D. T. (2002). Cooperative fishing interactions between Aboriginal Australians and dolphins in eastern Australia. *Anthrozoös*, *15*(1), 1–18. https://doi.org/10.2752/089279302786992694

Nelson, R. (1983). *Make Prayers to the Raven*. University of Chicago Press.

Orams, M. B. (1997). Historical accounts of human-dolphin interaction and recent developments in wild dolphin based tourism in Australasia. *Tourism Management*, *18*(5), 317–326. https://doi.org/10.1016/S0261-5177(96)00022-2

Ovodov, N. D., Crockford, S. J., Kuzmin, Y. V., Higham, T. F. G., Hodgins, G. W. L., & van der Plicht, J. (2011). A 33,000-year-old incipient dog from the Altai Mountains of Siberia: evidence of the earliest domestication disrupted by the last Glacial Maximum. *PLoS ONE*, *6*(7), 4–10. https://doi.org/10.1371/journal.pone.0022821

Pellegrini, A. Y., Romeu, B., Ingram, S. N., & Daura-Jorge, F. G. (2021). Boat disturbance affects the acoustic behaviour of dolphins engaged in a rare foraging cooperation with fishers. *Animal Conservation*, 1–13. https://doi.org/10.1111/acv.12667

Peterson, D., Hanazaki, N., & Simões-Lopes, P. C. (2008). Natural resource appropriation in cooperative artisanal fishing between fishermen and dolphins (*Tursiops truncatus*) in Laguna, Brazil. *Ocean and Coastal Management*, *51*(6), 469–475. https://doi.org/10.1016/j.ocecoaman.2008.04.003

Pierotti, R., & Fogg, B. R. (2017). *The first domestication: how wolves and humans co-evolved*. Yale University Press.

Pliny the Elder. A.D. 23-79. (1940). *Natural History (H. Rackman, Trans.)*. Harvard University Press.

Pryor, K., & Lindbergh, J. (1990). A dolphin-human fishing cooperative in Brazil. *Marine Mammal Science*, *6*(1), 77–82.

Queeny, E. M. (1952). The Wandorobo and the honeyguide. *Journal of Natural History*, *6*, 392–396.

Ridgway, B. S. (1970). Dolphins and dolphin-riders. *Archaeology*, *23*, 86–95.

Righetti, B. P. H., Mattos, J. J., Siebert, M. N., Daura Jorge, F. G., Bezamat, C., Fruet, P. F., Genoves, R. C., Taniguchi, S., da Silva, J., Montone, R. C., Simões-Lopes, P. C., Bainy, A. C. D., & Lüchmann, K. H. (2019). Biochemical and molecular biomarkers in integument biopsies of free-ranging coastal bottlenose dolphins from southern Brazil. *Chemosphere*, *225*, 139–149. https://doi.org/10.1016/j.chemosphere.2019.02.179

Robinson, R. E. (1965). *The man who sold his dreaming*. Currawong Publishing Co Pty Ltd.

Romeu, B., Cantor, M., Bezamat, C., Simões-Lopes, P. C., & Daura-Jorge, F. G. (2017). Bottlenose dolphins that forage with artisanal fishermen whistle differently. *Ethology*, *123*(12), 906–915. https://doi.org/10.1111/eth.12665

Santos-Silva, B., Hanazaki, N., Daura-Jorge, F. G., & M., C. (2022). Social foraging can benefit artisanal fishers who interact with wild dolphins. *Behavioural Ecology and Sociobiology*, *76*, 42. https://doi.org/https://doi.org/10.1007/s00265-022-03152-2

Santos, M. L., Lemos, V. M., & Vieira, J. P. (2018). No mullet, no gain: cooperation between dolphins and cast net fishermen in southern brazil. *Zoologia*, *35*, 1–13. https://doi.org/10.3897/zoologia.35.e24446

Serpa, N. B. (2019). *Além das raízes culturais: as habilidades de caça de golfinhos cooperativos do sul do Brasil, Porto Alegre*. MSC thesis, Universidade Federal do Rio Grande do Sul.

Shipman, P. (2015). *The Invaders: How Humans and Their Dogs Drove Neanderthals to Extinction*. Harvard University Press.

Shternberg, L. A. (1933). *The Gilyaks, Orochi, Negidal, Ainu: Articles and Materials*. Dal'gi.

Silva, E., da Silveira, F. L. A., Marques, O. R., & Moreno, I. B. (2021). "A gente acostuma os olhos": pescadores artesanais de tarrafa e botos-de-Lahille nas paisagens da Barra do Rio Tramandaí. *Desenvolvimento e Meio Ambiente*, *56*, 22–45. https://doi.org/10.5380/dma.v56i0.72636

Simões-Lopes, P. C. (1991). Interaction of coastal populations of *Tursiops truncatus* (Cetacea, Delphinidae) with the mullet artisanal fisheries in Southern Brazil. *Biotemas*, *4*(2), 83–94.

Simões-Lopes, P. C., Daura-Jorge, F. G., & Cantor, M. (2016). Clues of cultural transmission in cooperative foraging between artisanal fishermen and bottlenose dolphins, *Tursiops truncatus* (Cetacea: Delphinidae). *Zoologia*, *33*(6), e20160107. https://doi.org/10.1590/S1984-4689zool-20160107

Simões-Lopes, P. C., Fabián, M. E., & Menegheti, J. O. (1998). Dolphin interactions with the mullet artisanal fishing on southern Brazil: a qualitative and quanititative approach. *Revta Bras. Zool.*, *15*(3), 709–726.

Skead, C. J. (1951). Notes on Honeyguides in Southeast Cape Province, South Africa. *The Auk*, *68*(1), 52–62.

Smith, B. D., Than, M., Myo, A., Win, H., & Moe, T. (2009). Catch composition and conservation management of a human-dolphin cooperative cast-net fishery in the Ayeyarwady River, Myanmar. *Biological Conservation*, *142*(5), 1042–1049. https://doi.org/10.1016/j.biocon.2009.01.015

Sparrman, A. V. (1777). An account of a journey into Africa from the Cape of Good-Hope, and a description of a new species of cuckow. By Dr. Andreas Sparrman, of the Royal Academy of Stockholm, in a letter to Dr. John Reinhold Forster, F.R.S. Philosophical Tr. *Transactions of the Royal Society of London*, *67*, 38–47.

Spottiswoode, C. N., Begg, K. S., & Begg, C. M. (2016). Reciprocal signaling in honeyguide-human mutualism. *Science*, *353*(6297), 387–389. https://doi.org/10.1126/science.aaf4885

Standing Bear, L. (1978). *Land of the Spotted Eagle*. University of Nebraska Press.

Stebbins, E. B. (1929). *The dolphin in the literature and art of Greece and Rome.* George Banta Publishing Company.

Thein, U. T. (1977). The Burmese freshwater dolphin. *Mammalia*, *42*(2), 233–234.

Tun, T. (2004). *Irrawaddy Dolphins in Hsithe - Mandalay segment of the Ayeyawady River and cooperative fishing between Irrawaddy Dolphin,* Orcaella brevirostris*, and castnet fishermen in Myanmar. Report submitted to Wildlife Conservation Society*.

Tun, T. (2005). *Castnet Fisheries in Cooperation with Irrawaddy Dolphins (Ayeyawady Dolphins) at Hsethe, Myitkangyi and Myayzun Villages, Mandalay Division, in Myanmar. Report submitted to Wildlife Conservation Society.*

Tun, T. (2014). Castnet fishing with the help of Irrawaddy Dolphin, *Orcaella brevirostris*, in Myanmar. *Proceedings of the Design Symposium on Conservation of Ecosystem (The 13th SEASTAR2000 Workshop)*, *2*, 39–45.

Turgut, M. (2010). The myth of youth Hermias and his dolphin at Iasos in Caria. *Child's Nervous System*, *26*(4), 407–409.

van der Wal, J. E. M., Gedi, I. I., & Spottiswoode, C. N. (2022). Awer honey-hunting culture with greater honeyguides in coastal Kenya. *Frontiers in Conservation Science*, *2*, 727479. https://doi.org/10.3389/fcosc.2021.727479

Ville, J. L. (1995). The Waata of Tsavo-Galana: hunting and trading in their semi-arid coastal hinterland. *Kenya Past and Present*, *27*(1), 21–27.

Walker, B. L. (2005). *The Lost Wolves of Japan*. University of Washington Press.

Wang, J. Y., Costa, A. P., & Jefferson, T. A. (2021). The correct name of Lahille's bottlenose dolphin, *Tursiops truncatus gephyreus* Lahille, 1908. *Marine Mammal Science*, *37*(2), 696–701.

Westdal, K. H., Higdon, J. W., & Ferguson, S. H. (2017). Attitudes of Nunavut Inuit toward Killer Whales (Orcinus orca). *Arctic*, *66*(3), 279–290.

Wickert, J. C., von Eye, S. M., Oliveira, L. R., & Moreno, I. B. (2016). Revalidation of *Tursiops gephyreus* Lahille, 1908 (Cetartiodactyla: Delphinidae) from the southwestern Atlantic Ocean. *Journal of Mammalogy*, *97*(6), 1728–1737.

Wood, B. M., Pontzer, H., Raichlen, D. A., & Marlowe, F. W. (2014). Mutualism and manipulation in Hadza-honeyguide interactions. *Evolution and Human Behavior*, *35*(6), 540–546. https://doi.org/10.1016/j.evolhumbehav.2014.07.007

Zappes, C. A., Andriolo, A., Simões-lopes, P. C., & Di Beneditto, A. P. M. (2011). 'Human-dolphin (*Tursiops truncatus* Montagu, 1821) cooperative fishery' and its influence on cast net fishing activities in Barra de Imbé/Tramandaí, Southern Brazil. *Ocean & Coastal Management*, *54*, 427–432. https://doi.org/10.1016/j.ocecoaman.2011.02.003
